# High definition video teaching module for learning neck dissection

**DOI:** 10.1186/1916-0216-43-7

**Published:** 2014-03-25

**Authors:** Adrian Mendez, Hadi Seikaly, Kal Ansari, Russell Murphy, David Cote

**Affiliations:** 1 Division of Otolaryngology, Head and Neck Surgery, University of Alberta, Edmonton, Alberta, Canada; 2 University of Alberta Hospital, 8440-112 Street, Room 1E4, WMC, Edmonton, Alberta T6G 2B7, Canada

## Abstract

**Introduction:**

Video teaching modules are proven effective tools for enhancing student competencies and technical skills in the operating room. Integration into post-graduate surgical curricula, however, continues to pose a challenge in modern surgical education. To date, video teaching modules for neck dissection have yet to be described in the literature.

**Purpose:**

To develop and validate an HD video-based teaching module (HDVM) to help instruct post-graduate otolaryngology trainees in performing neck dissection.

**Methods:**

This prospective study included 6 intermediate to senior otolaryngology residents. All consented subjects first performed a control selective neck dissection. Subjects were then exposed to the video teaching module. Following a washout period, a repeat procedure was performed. Recordings of the both sets of neck dissections were de-identified and reviewed by an independent evaluator and scored using the Observational Clinical Human Reliability Assessment (OCHRA) system.

**Results:**

In total 91 surgical errors were made prior to the HDVM and 41 after exposure, representing a 55% decrease in error occurrence. The two groups were found to be significantly different. Similarly, 66 and 24 staff takeover events occurred pre and post HDVM exposure, respectively, representing a statistically significant 64% decrease.

**Conclusion:**

HDVM is a useful adjunct to classical surgical training. Residents performed significantly less errors following exposure to the HD-video module. Similarly, significantly less staff takeover events occurred following exposure to the HDVM.

## Background

Modern surgical training has evolved to incorporate simulation and surgical skills laboratories in addition to traditional operating room teaching [1]. Many *ex vivo* surgical learning models have been employed including virtual reality simulators, bench top simulators, and video modules. Many of these types of surgical learning modules have been validated for effectiveness in randomized controlled trials as well as systematic reviews [1].

The current curriculum for modern surgical training includes both *technical skills training* (TST) as well as *cognitive training* (CT). TST refers to procedural technique training while CT refers to understanding the steps and sequence of a procedure. The importance of cognitive training has been highlighted in a study analyzing common surgical errors made by training residents where 60 laparoscopic cholecystectomies performed by surgical trainees were video recorded and analyzed for errors. 1067 total errors were observed with 2 out of the 3 most common errors being attributed to erroneous surgical steps [2].

The graduating otolaryngology-head and neck surgery resident is expected to be capable of performing several surgical procedures requiring capacity in surgical skill as well as cognitive prowess in knowing appropriate steps for carrying out the procedure safely and effectively. Among such procedures is arguably the most cognitively complex – the neck dissection. Often a learning objective of the senior Otolaryngology-Head and Neck Surgery resident, it is a skill that is made difficult from the variety, number, and order of surgical steps. A review of the literature indicates no previously published and validated simulation or video modules in neck dissection instruction.

The purpose of this study was to develop and validate a high definition video-based teaching module to facilitate cognitive training of post-graduate Otolaryngology-Head and Neck Surgery trainees in performing a unilateral neck dissection.

## Methods

### Study design

This was a prospective study conducted at a tertiary care academic head and neck surgery referral center. Ethics approval was obtained from the health research ethics board at the University of Alberta in Edmonton, Alberta [Pro00036896].

### High definition video module

The high definition video module (HDVM) was created using three camera angles all shot in High Definition: a handheld camera, an overhead surgical camera, and a surgical headlight camera (Integra® DLX Ultralite® Pro Camera). The video captured a head and neck surgical attending at the University of Alberta performing a level II-IV selective neck dissection. The edited video module is 14 minutes in length. The video includes added text clearly outlining the surgical steps in sequence with the video. Participants also received a more detailed document of the consecutive surgical steps performed, which were numbered to match those of the video. The content validity of the teaching materials was ensured through a peer review process with five head and neck surgeons and using historically published technical protocols. Understandability was confirmed using a focus group of first and second year Otolaryngology-Head and Neck Surgery residents at the University of Alberta.

### Participants

Inclusion criteria for the study were Otolaryngology-Head and Neck Surgery residents in post-graduate year 3 through 5 at the University of Alberta. Participants were recruited by e-mail and participation was voluntary. Informed consent was obtained, ensuring that the study would have no impact on their formative and summative assessments for promotion or advancement in their residency training. Of those approached 100% enrollment was seen and all subjects completed the study.

### Initial assessment (pre video module)

After enrollment into the study, participants were given a date for their initial assessment. They were instructed to perform a unilateral selective neck dissection (levels II-IV) on a radiographic N0 neck. While performing the neck dissection, participants wore an Integra® DLX Ultralite® Pro Camera in order to video record the procedure. All participants were de-identified and wore white surgical gloves and the attending staff surgeon wore brown surgical gloves to aid with evaluation of the video by a separate blinded reviewer. The video included no audio. A member of the study team was also present at all times during the procedures to facilitate recording and to document any ‘take over’ events by the staff surgeon.

### Final assessment (post video module)

Following a washout period of at least 2 weeks after the initial assessment to eliminate carry-over effect, participants were given the HDVM the day prior to their final assessment. The following day, participants again performed a unilateral selective neck dissection (levels II-IV) on a radiographic N0 neck. The procedure was video recorded using the Integra® DLX Ultralite® Pro Camera.

### Evaluation

Assessment of technical skill must be objective, reliable, and easy to perform. Current systems for this type of assessment can be divided into *dexterity-based* and *video-analysis* systems [3]. Dexterity-based systems record parameters including path length, number of movements, and instrument trajectories. Video analysis provides a qualitative method of assessment. However, quantification of performance through definition of correct steps and errors, although labor intensive, is a powerful mode of both *formative* and *summative* assessment [4]. This entails development of a procedure-specific scale, referred to as *task analysis*, which is an amalgamation of tasks and subtasks. Dexterity analysis is generic but video-based analysis can be used in this procedure-specific manner.

For this study, we used the Observational Clinical Human Reliability Assessment (OCHRA) system to assess surgical performance, a system previously validated to assess the quality of surgical operative performance by documenting the errors and the stage of the operation in which errors are enacted most frequently [5]. Error identification was based on 10 generic forms of error (Table 1) that can be predicted for the execution of a surgical task. These 10 types of errors represent observed patterns of failure and fall in 2 categories based on their causative mechanism. Errors 1 through 6 have to do with the ability of the surgeon to perform the component steps of the operation in the correct sequence and are grouped as *Procedural Errors*. Errors 7 through 10 reflect manipulation of instruments in performing a specific task and are referred to as *Execution Errors*.

**Table 1:** Categorization of errors

**Pattern of failure**	**Definition**
1	Step is not done
2	Step is partially completed
3	Step is repeated
4	Second step is done in addition
5	Second step is done instead of first step
6	Step is done out of sequence
7	Step is done with too much force or speed
8	Step is done with too little force or speed
9	Step is done in wrong orientation or direction
10	Step is done on/with the wrong object

A task analysis of a selective neck dissection used at the University of Alberta was undertaken, using a videotaped procedure, the historical technical protocol of the procedure [6], as well as input from five head and neck surgeons at the University of Alberta. The task analysis (Table 2) divided the procedure into 15 ‘*generic*’ tasks, which could be found within most selective neck dissection procedures. This allowed the comparison of error rates between tasks within the procedure as well as making it possible to expand the utilization of the task analysis more widely to other forms of selective neck dissections performed at other surgical centers.

**Table 2:** Task analysis

**Sequence of tasks**	**Generic task**	**Sub-tasks**
1	Find the MM nerve	1.1 Use landmarks
		1.2 Cut parallel to nerve with scalpel
		1.3 Trace retrograde
2	Dissect external jugular vein from anterior border of SCM	
3	Unwrap the SCM	3.1 Grasp SCM in nondominant hand, retracting laterally
		3.2 Run monopolar along edge of SCM
		3.3 Roll SCM laterally
4	Identify CN XI	
5	Identify posterior belly of digastric	5.1 Identify posterior edge of submandibular gland
		5.2 Identify digastric tendon using army navy and dental
		5.3 Dissect superficial to the muscle in posterior direction
6	Skeletonize CN XI	
7	Expose carpet/floor of neck	7.1 Identify IJV
		7.2 Dissect superior aspect of IJV
		7.3 Find carpet in the lateral posterior border of jugular vein
8	Release level IIb	8.1 Monopolar or bipolar scissors to remove fibrofatty tissue from level IIB
		8.2 Dunk under CN XI
9	Free nodal packet from IJV inferiorly	9.1 Identify the IJV
		9.2 Dissect nodal packet free off IJV
		9.3 Cut fascia posterior to jugular vein
		9.4 Use dentals to sweep nodal packet superiorly
		9.5 Identify phrenic
		9.6 Cut omohyoid as goes into level V of neck
10	Dissect out and preserve cervical rootlets	10.1 Use 15blade and run parallel to posterior SCM
		10.2 Identify cervical rootlets
		10.3 Change to plane medial to rootlets
11	Identify the vagus nerve	11.1 Identify carotid sheath
		11.2 Identify vagus nerve
		11.3 Cut on vagus in a parallel plane
12	Free the IJV	12.1 Incise sheath over IJV
		12.2 Cut parallel to “white line”
		12.3 Identify and preserve any branches of IJV
13	Hypoglossal nerve	13.1 Identify hypoglossal nerve with blunt dissection or dental anterior to jugular vein
		13.2 Release nodal pack superior to inferior preserving lingual vein and hypoglossal nerve
14	Dissect down to strap muscles	14.1 Identify ansa
		14.2 Cut medial to ansa
		14.3 Identify and preserve superior thyroid artery and veins
		14.4 Continue and identify strap muscles
15	Free nodal package from straps and digastric	15.1 use monopolar cautery to free up

### Data analysis

This study recorded 12 selective neck dissections. These operations were performed by 6 Otolaryngology-Head and Neck Surgery trainees in PGY 3–5 who performed 2 selective neck dissections each, pre and post video module exposure. All operations were done under the supervision of the same head and neck staff surgeon at the University of Alberta who was blinded to the type of evaluation. The anonymously recorded procedures were then sent to a head and neck fellowship trained independent evaluator in a random order using a random number generator. The evaluator was blinded to the participant and whether they have been exposed to the video module. The evaluator then allocated each observed error to the step it occurred and categorized it as a *Procedural* or *Execution Error*. If it was possible to attribute a single error event to more than one pattern of failure (as outlined in Table 1), this was still only counted as a single error when tabulating the results. The evaluator also viewed and utilized the HDVM as the benchmark for gauging errors. The Mann–Whitney *U* test was used to calculate the difference in number of errors made and staff takeover events between the pre and post module groups. A *p* value of less than 0.05 was considered significant.

## Results

**Figure 1:**
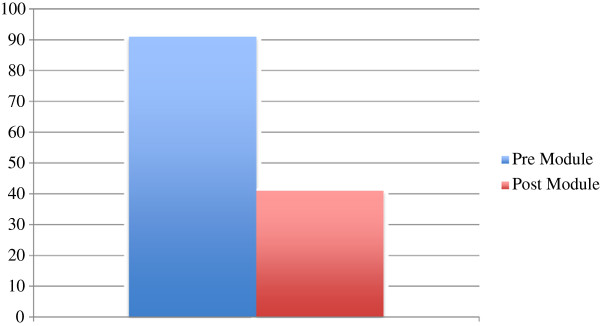
Total error aggregate.

**Figure 2:**
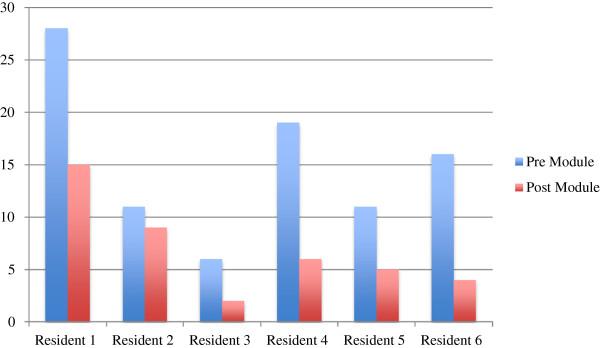
Total error stratified per resident.

**Figure 3:**
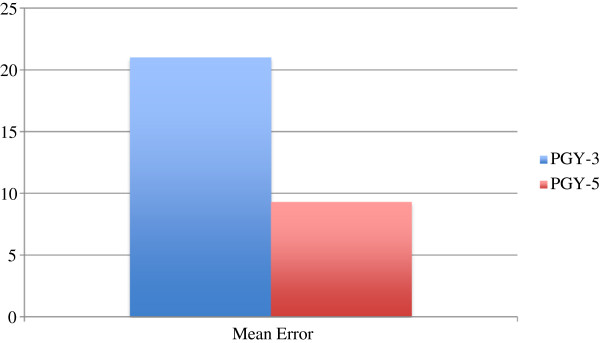
Mean error comparison between PGY-3 and PGY-5.

In total 6 Otolaryngology-Head and Neck Surgery residents at the University of Alberta were included in the study (Table 3). The total numbers of errors committed prior to exposure to the HDVM were 91. The total numbers of errors committed following exposure to the module were 41. This represented a 55% decrease in the number of errors committed pre-HDVM that was statistically significant (Figure 1, Table 4). Figure 2 stratifies these total numbers of errors per individual resident in the study. Figure 3 compares the mean number of errors committed by the PGY-5 group and PGY-3 group and shows that on average, the PGY-5 group performs less surgical errors than the counterpart.

**Table 3:** Participants and year of training

**Post graduate year**	**N = 6**
PGY 3	3
PGY 4	0
PGY 5	3

**Table 4:** Outcome measures

**Outcome measured**	**Pre module median (n = 6)**	**Post module median (n = 6)**	**Pre module mean (n = 6)**	**Post module mean (n = 6)**	**P-value**
# of errors	13.5	5.5	15.2	6.8	<0.05
# of staff takeovers	5.5	3	11	4	<0.05

**Figure 4:**
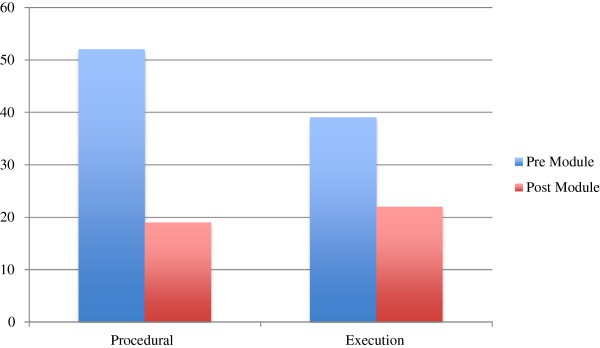
Types of error.

Figure 4 separates the error types and compares them before and after exposure to the HDVD. We see that the number of *Procedural Errors* are reduced from 52 to 19 after exposure to the HDVD. Similarly the number of *Execution Errors* are reduced from 39 to 22 after exposure to the HDVM.

**Figure 5:**
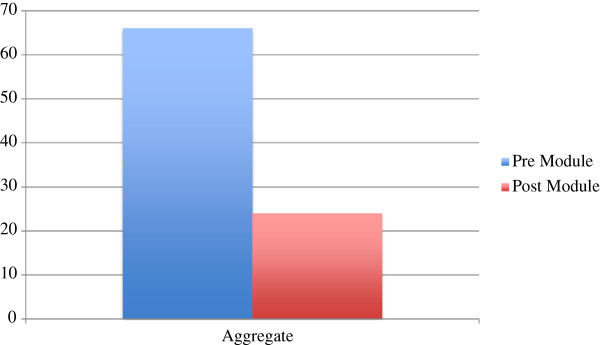
Total staff takeover events.

**Figure 6:**
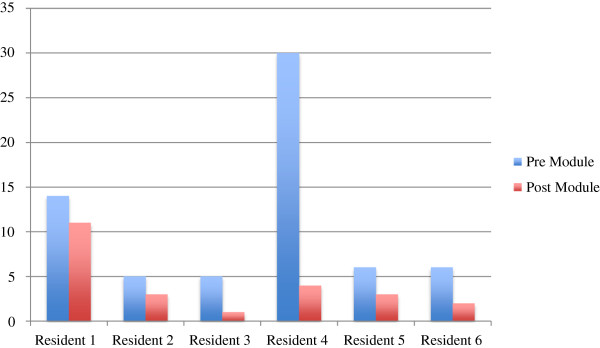
Staff takeover events per resident.

The total number of ‘take over’ events by the surgical attending before and after exposure to the HDVM was 66 and 24 respectively (Figure 5). This represented a 64% decrease following exposure to the HDVM that was statistically significant (Table 4). Figure 6 stratifies these results per individual resident in the study.

## Discussion

Educators are increasingly seeking interventions in medical education that objectively produce measurable improved training outcomes of the highest order. Surgical education poses specific challenges as it involves both *cognitive* and *technical* training. Recent focus in surgical education has turned to quantifying error events as a means of analyzing surgical performance in trainees in addition to outcomes and complications [7]. This system allows objective tracking of errors related to the performance of a specific operation.

Surgical error analysis allows identification of not only the quantity of errors being committed but also during what portion of the procedure they are occurring (*Hazard Zones*) and the underlying causative mechanism [2,5]. This information is important as it can aid in attempting to correct these errors in learners and indicate to the attending which portions of the procedure are particularly vulnerable to surgical error. This study, for example, indicated that errors were most likely to occur during dissection of the internal jugular vein. The OCHRA system used to evaluate surgical performance in this study is based on *industrial human reliability assessment* and has been shown by Tang *et al.* to be able to be adapted for clinical use to provide a comprehensive objective assessment of performance of a specific operative (open or endoscopic) procedure [5,7].

There may also be an increasing need for *ex vivo* models for surgical skills training as regulations regarding resident duty hour restrictions continue to be imposed, thereby limiting the hours of clinical training. Furthermore, in an economically challenged climate, operative resources are being increasingly curtailed, forcing surgeons to be more efficient with both performing and teaching operations. Medico-legally speaking, the public demands the highest level of preparation and safety during operative procedures. To respond to these forces, *ex vivo* models to prepare residents to be safer and more efficient in operating room is a very viable and increasingly necessary solution. This model is also an excellent training adjunct for competency based medical education for not only teaching a surgical procedure but also evaluating and providing high quality feedback to the learner - a high priority initiative of the Royal College of Physicians and Surgeons of Canada. This study sought to validate a learning module to teach arguably one of the most complex and involved surgical procedures Otolaryngology-Head and Neck Surgical residents must learn during their training.

The results do show improvement following exposure to the HDVM. The validity of our approach in also demonstrated in the initial control neck dissections where resident trainees in higher years of training committed less surgical errors than those in lower years of training giving our method of evaluation construct validity (Figure 3). Also of note is that senior resident trainees in PGY-5 still showed improvement following exposure to the HDVM, indicating that even trainees who have potentially performed several dozen neck dissections can benefit from the module.

This study also sought to identify what types of errors (*Procedural* or *Execution*) were being more commonly committed by surgical residents and we found that overall, similar numbers of each type of errors were being committed. *Procedural Errors* refer to cognitive type errors (knowledge of correct surgical steps, sequence, etc.) while *Execution Errors* refer to technical type errors (using the correct amount of force, speed, etc.). Interestingly, this is in contrast to recent literature, which indicates that surgical residents primarily commit cognitive type errors [7].

As otolaryngology programs are typically small, our sample size was also small, limiting the generalizability of our results. Future multi-institutional collaboration may be considered to overcome this limitation as well as being able to ascertain higher order medical education outcomes such as accelerated acquisition of learner competence for the procedure (thereby reducing their overall training time), reduced operating room time and cost, and reduction in patient morbidity and mortality. In addition, as our learners were only evaluated 24 hours after our intervention, we cannot gage long-term retention. There may be value in repeating evaluation 6 months post-intervention to determine if surgical error reduction was sustained.

## Conclusion

HDVM is a useful adjunct to classical surgical training. Otolaryngology-Head and Neck Surgery trainees at the University of Alberta show reduction of the number of errors committed and reduction of staff takeover events during neck dissection following exposure to the HDVM.

## Competing interests

The authors declare that they have no competing interests.

## Authors’ contributions

AM participated in the design of the study, performed statistically calculations, and drafted the manuscript. HS participated in the design of the study, participated in all surgical procedures in the study. KA participated in the design of the study, and evaluated all surgical videos in the study. RM participated in the surgical procedures in the study as well as coordination of the study. DC conceived the study and participated in the design of the study. All authors read and approved the manuscript.
